# MUC1-Specific Cytotoxic T Lymphocytes in Cancer Therapy: Induction and Challenge

**DOI:** 10.1155/2013/871936

**Published:** 2012-12-26

**Authors:** David Roulois, Marc Grégoire, Jean-François Fonteneau

**Affiliations:** ^1^UMR892, INSERM, Institut de Recherche Thérapeutique, Université de Nantes, 8 quai Moncousu, BP70721, 44007 Nantes Cedex 1, France; ^2^CNRS, UMR6299, Institut de Recherche Thérapeutique, Université de Nantes, 8 quai Moncousu, BP70721, 44007 Nantes Cedex 1, France; ^3^Faculté de Médecine, Université de Nantes, 44035 Nantes Cedex 1, France

## Abstract

MUC1 glycoprotein is often found overexpressed and hypoglycosylated in tumor cells from numerous cancer types. Since its discovery MUC1 has been an attractive target for antitumor immunotherapy. Indeed, *in vitro* and *in vivo* experiments have shown T-cell-specific responses against MUC1 in an HLA-restricted and HLA-unrestricted manner, although some animal models have highlighted the possible development of tolerogenic responses against this antigen. These observations permit the development of new T-cell vaccine strategies capable of inducing an MUC1-specific cytotoxic T cell response in cancer patients. Some of these strategies are now being tested in clinical trials against different types of cancer. To date, encouraging clinical responses have been observed with some MUC1 vaccines in phase II/III clinical trials. This paper compiles knowledge regarding MUC1 as a promising tumor antigen for antitumor therapeutic vaccines applicable to numerous cancers. We also summarize the results of MUC1-vaccine-based clinical trials.

## 1. Introduction

With the increasing number of cancers, the development of innovative cancer therapies is a great challenge. One of these innovative strategies is immunotherapy. Since the discovery that the immune system can control cancer progression, which has been conceptualized in the “three Es” theory [1] for “elimination, equilibrium, and escape,” supporting the implications of the immune system in the control and selection of tumor cells, scientists and clinicians have tried to exploit this phenomenon to induce an antitumor immune response in cancer patients. Major goals in the field of immunotherapy are to understand how the immune system can be specifically activated against cancer cells and to identify relevant antigenic cancer targets.

The first human tumor-associated antigen (TAA) to be discovered, recognized by cytotoxic CD8+ T lymphocytes (CTL), was MAGE-A1 which was identified from tumor-infiltrating lymphocytes obtained after culture of a melanoma biopsy [2]. Since then, many other TAAs have been identified (for review see [3]). Certain TAAs are restricted to one or a few cancer types, such as the “mutated TAA” (BCR-ABL fusion, B-raf, k-ras, N-ras, p53, etc.), or the “differentiation TAA” (Melan-A/MART1, gp100, CEA, PSA, etc.), whereas others are shared between a wide range of cancers, such as the “shared tumor-specific TAA” (MAGE, NYESO-1, SSX, etc.) or the “overexpressed TAA” (HER-2/Neu, p53, Telomerase, MUC1, etc.) (http://www.archive.cancerimmunity.org/peptidedatabase/Tcellepitopes.htm).

Mucin 1 (MUC1) belongs to the “overexpressed TAA” category, even if this overexpression is not the only hallmark of MUC1 in tumor cells, since it is often accompanied by modification of MUC1 glycosylation status. In healthy cells, MUC1 is a glycoprotein expressed at the apical surface of epithelial cells and characterized by a high glycosylation level. In cancers, this glycoprotein is often overexpressed by tumor cells, with a loss of polarity and, interestingly, a modification of its glycosylation pattern [4]. Both the overexpression and the modification of its glycosylation status make this protein highly immunogenic and, thus, an interesting target in cancer immunotherapy. In this paper, we focus on the immunogenic properties of the MUC1 glycoprotein. However, it should be noted that MUC1 has also been described as having an oncogenic role (for review see: [5, 6]). Firstly, we describe the differences between MUC1 expression in healthy cells and tumor cells which renders MUC1 more immunogenic when it is expressed by tumor cells. Secondly, we discuss the problem of induction of tolerance against MUC1 which can impair the antitumor immune response. We list the immunotherapy strategies for inducing an antitumor response in patients, which are being developed *in vitro* and in mouse models. Finally, we discuss MUC1-based immunotherapy clinical trials against cancers.

## 2. MUC1: An Overexpressed, Hypoglycosylated, Tumor-Associated Antigen

**Figure 1:**
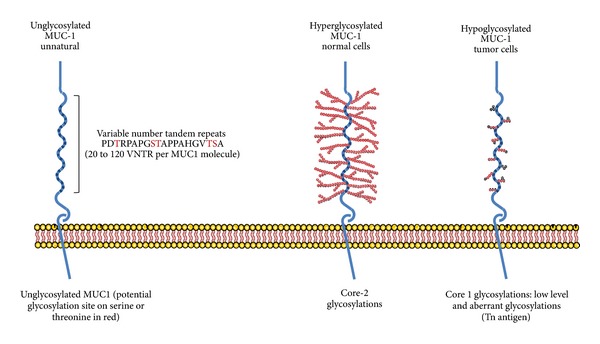
Structure of the MUC1 glycoprotein in normal and tumor cells.

The *MUC1* gene was cloned in the early 1990s [7, 8]. It belongs to the mucin family, comprising 21 members. *MUC1* encodes a highly glycosylated, type I transmembrane glycoprotein, with a variable number of 20-amino-acid repeat sequences referred to as “variable number tandem repeat” (VNTR) (Figure 1). The number of VNTR is variable from one allele to another, varying from 25 to 120 VNTR per MUC1 molecule, with the alleles containing 40 and 66 VNTR being the most frequent in the northern European population [7]. Each VNTR contains five potential sites of O-glycosylation on serine or threonine.

Towards the end of the 1980s, differences between the MUC1 expressed by healthy mammary cells and by breast cancer cells were observed using the monoclonal antibody, SM-3, specific for the MUC1 core protein stripped of sugars [9, 10]. Indeed, SM-3 mAb used in histology recognized 91% of breast cancer samples, but showed little or no reactivity with healthy mammary cells [9]. The SM-3 mAb was also reactive against lung, colon, and ovarian carcinoma, but failed to stain the healthy cell counterparts [10]. These studies showed that, in different types of carcinoma, MUC1 is hypoglycosylated. This hypoglycosylation allows recognition by the SM-3 mAb. Since then, MUC1 was considered to be a “tumor-associated antigen” which can be targeted in immunotherapy using monoclonal antibodies. It has subsequently been shown that the glycosylation structures of MUC1 expressed by normal breast cells and tumor cells are different. Indeed, in healthy cells MUC1 contains extended, core 2-based glycans that are formed by N-acetylglucosamine attachment to the GalNAc of core 1, while on the MUC1 expressed by tumor cells the glycans are shorter, core 1-based and richer in ST, Tn, and T glycans [11–13].

These differences observed between MUC1 expressed by tumor cells and healthy cells prompted research on the capacity of T lymphocytes to recognize tumoral MUC1 epitopes. T lymphocytes usually recognize, with their T-cell receptors, peptides from endogenous or exogenous antigens presented in association with MHC molecules. Surprisingly, the first report of recognition of MUC1 by T lymphocytes was shown to be non-MHC-restricted [14]. Indeed the T lymphocytes obtained from a pancreatic cancer patient did not recognize an MUC1 peptide presented by an MHC molecule, but rather, directly, the hypoglycosylated core of MUC1. This unusual antigen recognition was confirmed by other groups [15–18]. The reactivity of these T cells was inhibited by SM-3 mAb, which showed that these T cells recognize the hypoglycosylated core of MUC1. Furthermore, these T cells failed to recognize healthy epithelial cells, which were not stained by the SM-3 mAb. Finally, Hinoda and colleagues described an increased recognition of gastric tumor cells cultured with benzyl-2-acetamido-2-deoxy-*α*-D-galactopyranoside (BGN), a competitive inhibitor of O-glycosylation, by an HLA-unrestricted, MUC1-specific CTL line [19]. All of these results suggest that a non-MHC-restricted, hypoglycosylated, MUC1-specific T-cell response can be present spontaneously in cancer patients.

In the mid 1990s, efforts were made to identify HLA-restricted, MUC1-specific T-cell responses. Indeed, progress made in the understanding of the T-lymphocyte response against tumor cells allowed the development of new strategies to identify TAA, such as “reverse immunology”. This consists of inducing *in vitro* T-lymphocyte responses against peptides from a candidate TAA. Peptides are selected for their capacity to bind a particular HLA allele. The capacity of peptide-responding T cells to recognize a tumor cell line which expresses the candidate TAA and the particular HLA allele is then tested to validate the epitope. Using this approach, Domenech and colleagues identified a peptide encoded by the VNTR (STAPPAHGV) with the ability to bind to several HLA class I alleles: HLA-A1, -A2.1, -A3, and -A11 [20]. They were able to generate cytotoxic CD8+ T lymphocytes specific for HLA-A11/STAPPAHGV, but did not validate the presentation of this peptide by HLA-A11+ tumor cells. Using HLA-A*0201/Kb transgenic mice, Apostolopoulos and colleagues identified two peptides from the VNTR able to bind HLA-A*0201 molecule, which are the most common HLA class I allele in the Caucasian population: the peptide, STAPPAHGV, previously described by Domenech's team, and a new peptide, APDTRPA [21]. These peptides were able to induce CD8+ cytotoxic T-cell responses in HLA-A*0201/Kb mice. The T cells were able to lyse the HLA-A*0201+ MUC1+ breast cancer cell line, MCF-7. Brossart and colleagues then selected two other peptides from MUC1: MUC1(20–28) LLLLTVLTV and MUC1(950–958) STAPPVHNV, which exhibit a good affinity for the HLA-A*0201 molecule [22]. The STAPPVHNV peptide is not encoded by the VNTR, but by the region flanking it, whereas the LLLLTVLTV peptide is encoded by the signal sequence of MUC1. Brossart and colleagues generated two CD8+ T-cell clones specific for the HLA-A*0201/MUC1(20–28) and HLA-A*0201/MUC1(950–958) complexes. These clones were able to recognize MUC1+ HLA-A*0201+ tumor cell lines from different types of cancer: breast, pancreatic, and renal. In another study, the same researchers showed that these MUC1-specific T-cell clones were also able to recognize multiple myeloma cells and primary acute myelogenous leukemia blasts [23]. More recently, we showed that MUC1(950–958) peptide is presented to MUC1-specific CD8+ T cells by HLA-A*0201+ malignant pleural mesothelioma cell lines [24]. In addition, Ninkovic and colleagues reported that some glycosylated peptides from the VNTR of MUC1, notably the decamer SAP10 [SAPDT(GalNAc)RPAPG], can be generated by the immunoproteasome of dendritic cells [25]. This glycosylated peptide can be presented by HLA class I molecules and recognized by CD8+ T cells. The nonglycosylated peptide was also recognized by CD8+ T cells, whereas a peptide with a longer sugar chain (Gal-GalNac) did not bind the HLA-A*0201 molecule. Finally, MUC1 can also be recognized by CD4+ T lymphocytes. However, in this case, only one HLA-DR3-restricted epitope encoded by the VNTR has been identified by “reverse immunology” [26]. Unfortunately, the capacity for presentation by tumor cells was not tested since they do not express HLA class II molecules.

Regarding the recognition of MUC1 peptides by HLA-restricted T lymphocytes, the importance of the MUC1 glycosylation status is not as clear as in the case of HLA-unrestricted recognition of MUC1. We showed recently that the MUC1 glycosylation level does not affect the recognition of mesothelioma tumor cells by an HLA-A*0201/MUC1(950–958)-specific CD8+ T-cell clone [24]. Indeed, we observed that some tumor cell lines recognized by the CD8+ T-cell clone were weakly stained with the SM-3 antibody and another mAb specific for hypoglycosylated MUC1, VU-3-C6. Furthermore, MPM cell lines treated with benzyl-2-acetamido-2-deoxy-*α*-D-galactopyranoside (BGN), a competitive inhibitor of O-glycosylation, were not better recognized by the T-cell clone, despite an increased staining with the SM-3 and VU-3-C6 mAb. However, other HLA-restricted MUC1 epitopes seem to be dependent on the hypoglycosylation status of MUC1, at least for the induction of the MUC1-specific T-cell response by antigen-presenting cells such as dendritic cells (DC). Indeed, Hiltbold and Colleagues reported that glycosylation of long peptides, consisting of five MUC1 tandem-repeat regions, decreased the processing and the HLA-A1 restricted cross-presentation to CD8+ T cells by DC of a nine-amino-acid peptide contained in this long peptide [27]. Furthermore, the modification of MUC1 glycosylation in cancer cells may increase the capacity of DC to acquire this antigen for cross-presentation. Indeed, in cancer, soluble MUC1 presents an aberrant pattern of glycosylation, notably the Tn antigen, which can be recognized and internalized by the C-type lectin receptor macrophage galactose-type lectin (CLR MGL) present on DC and macrophages [28, 29]. This internalization of the soluble form of this tumoral form of MUC1 antigen by DC has also been shown to be mediated by the mannose receptor [30]. However, the retention of internalized MUC1 in the early endosome by this receptor inhibits its presentation to CD4+ T cells, whereas the nonglycosylated form of the antigen is well presented. Conversely, Vlad and colleagues reported that DC exposed to a long-glycosylated MUC1 peptide were able to process and present a glycosylated shorter peptide, in association with class II HLA, to CD4+ T cells suggesting that glycosylated MUC1 peptide can be processed by dendritic cells. [28]. In addition, it has been shown that the glycosylation status influences, but does not inhibit, the cleavage of MUC1 by the immunoproteasome expressed by mature DC [31]. All of these studies underline the capacity of dendritic cells (DC) to distinguish normal from aberrant MUC1. This shows the importance of MUC1 glycosylation, which affects differently the presentation of abnormal MUC1 in MHC class I and II molecules according to the MUC1 epitope studied.

## 3. Human MUC1 Mouse Model and Tolerance

With the goal of studying the immunogenicity of MUC1 *in vivo* and of developing MUC1-based cancer immunotherapy, human MUC1 transgenic mouse (TG mice) tumor models were set up by the group of Papadimitriou [32]. These authors reported that the expression of human MUC1 in TG mice was closely similar to that observed in human tissue. Since then, different teams have used these TG mice to study tolerance against MUC1 and how to induce *in vivo* an MUC1 tumor-specific T-cell response [33–35]. Rowse and colleagues compared tumor growth of MUC1+ tumors in TG or Wt mice [35]. They also compared the induction of an MUC1-specific humoral response after immunization with MUC1 peptide. MUC1+ tumors grew in TG mice, whereas they were rejected in Wt mice, suggesting that a tolerance to MUC1 is present in TG mice. MUC1 immunization of Wt mice also allowed a switch of immunoglobulin to the IgG subtype, whilst this switch was not observed in TG mice. Two other studies have also demonstrated the establishment of tolerance against MUC1 in TG mice [36, 37]. In a first study, the authors showed that a CD4+ T-cell response against MUC1 occurred in Wt mice following challenge with MUC1-expressing B16 tumor cells, whereas this response was absent in TG mice [37]. When CD4+ T cells were transferred from B16-bearing Wt mice to B16-bearing TG mice, an increase in survival was observed. Furthermore, Wt mice challenged with MUC1-expressing B16 tumors mounted an IgG response against MUC1, whereas this IgG response was absent in TG mice [36]. Human MUC1 TG mice have since been crossed with conditional-endometriosis transgenic mice, showing the presence of regulatory Foxp3+ T cells and antibodies against MUC1 during endometriosis, with potential implications for cancer progression [38]. All of these studies, thus, show that a certain degree of MUC1 tolerance is established *in vivo*. MUC1-based immunotherapy strategies should take into account this phenomenon.

Indeed, these *in vivo* studies highlight the potential problem of MUC1 tolerance in humans. MUC1 is naturally expressed, and tolerogenic responses can occur in patients leading to tumor escape against the immune system. The MUC1 TG mouse tumor model seems to back up the idea of tolerance against MUC1 in humans, but the modifications of MUC1 glycosylation observed in human cancer cells were not taken into account in these studies. *In vivo* investigations using vaccination against MUC1 with glycosylated or nonglycosylated peptide suggest that tolerance is not established against epitopes of MUC1 carrying a tumor-specific pattern of glycosylation, such as the Tn antigen [39–41]. Thus, abnormal glycosylation patterns can be recognized as foreign rather than selfantigen, which seems to be important in the induction of a specific T-cell response [39, 40]. MUC1 can, thus, be considered as an “abnormal selfantigen” when its glycosylation status is modified in tumor cells and this is an interesting property that could be exploited in immunotherapy [42].

## 4. Strategy to Induce MUC1-Specific T-Cell Responses

Different approaches have been developed to induce MUC1-specific T-cell responses in cancer patients. The majority of these are based on the capacity of DC to activate specific T-cell immune responses [53]. We have summarized these approaches in Table 1 and focus on the two most-studied strategies: MUC1-encoding nucleic acids or peptidic vaccination.

One approach consists of the utilization of MUC1 DNA as a vaccine [50, 52, 54]. This strategy has the advantage of activating different populations of lymphocytes, contrary to the use of MHC class I or class II peptides. In these studies, injections of MUC1 cDNA vaccine to tumor-bearing mice were able to induce tumor regressions. However, in two studies, the effector T cells were identified as CD8+ cytotoxic T cells [52, 54], whereas in the last one, the antitumor response was attributed to CD4+ T cells [50]. Studies are now being performed to improve this approach, notably by adding to the MUC1 cDNA a maturation signal for DC. Indeed, one approach consists of coupling MUC1 DNA with DNA corresponding to the expression of a heat shock protein (HSP70) which has already been reported to improve the stimulatory properties of DC [55]. In this study, MUC1/HSP70-coupled DNA increased the capacity of DC to induce effective cytotoxic T-cell responses and inhibited tumor cell growth [45]. Another strategy which has been developed to improve MUC1 cDNA vaccination consists of combining the vaccine with a tumor-cell-death inducer, such as a cDNA interfering with the expression of ANT2, a protein implicated in carcinogenesis [51]. CD8+ T-cell responses were more effective using this combination than with the use of MUC1 cDNA alone. To target MUC1 cDNA expression only to DC, *in vitro* transfection of human DC with MUC1 mRNA has also been developed [46]. These MUC1-mRNA-transfected DC were able to induce MUC1-specific CD8+ T lymphocytes that can kill pancreatic cancer cells. Finally, to improve the MUC1 cDNA strategies, another interesting study suggested the use of a modified MUC1 sequence, in order to prevent normal glycosylation of the MUC1 protein in dendritic and cancer cells [47].

Another vaccination strategy which has been studied preclinically, in mice, consists of the use of MUC1 peptide or glycopeptides. For this strategy, the use of glycopeptides with tumor-specific sugar motifs, such as the Tn antigen, seems to be more efficient than the use of normal peptides in eliciting an effective immune response [39, 40]. The MUC1 peptide can also be modified to increase its penetration into antigen-presenting cells, for example, the MUC1-MPA(11)P peptide which improved the induction of tumor regression in an animal model [48]. Targeting of MUC1 peptide to antigen-presenting cells can also be improved by adding oxidized (T-cell response, weak antibody level) or reduced mannan (T cell response, high antibody level) [41]. Another interesting approach was recently developed using a plant model which can produce MUC1 glycopeptides that are able to break tolerance in MUC1 Tg mice vaccinated with this peptide by the production of MUC1-specific antibodies [56]. Some long MUC1 peptides containing MHC class I and class II epitopes (21 mer) have also been developed to induce CD8+ and CD4+ T-cell responses [44]. CD8+ T-cell responses obtained in mice with the long peptide seem to be stronger than with an MUC1 9 mer peptide. Peptidic MUC1 vaccines have also been combined with other reagents to improve their efficiency. For example, MUC1 glycopeptides have been covalently associated with T-helper peptide and TLR2 agonist in a multimodal vaccine [49]. This vaccine strategy was reported to induce a high level of MUC1-specific antibodies and MUC1-specific cytotoxic T lymphocytes, which showed superior capability to prevent tumors growth in mice than unglycosylated peptides.

## 5. MUC1-Based Immunotherapy: Clinical Studies

Some of these preclinical strategies are now being assessed for MUC1-based immunotherapy in clinical trials for different types of cancers. More than sixty clinical trials interested in MUC1 protein are currently in progress (http://www.clinicaltrials.gov/). The majority of these are developing vaccination strategies against MUC1 to treat cancer. This large number of trials highlights the clinical interest in MUC1 vaccination. Only three clinical studies have reached the phase IIB/III stage. We have summarized in Table 2 the strategies which are currently being developed in these clinical trials. Some of these studies have shown a possible clinical effect of this vaccine in inducing an MUC1-specific T-cell response. Among them, some major strategies have emerged and reached phase III.

The BLP25 liposome vaccine (stimuvax or L-BLP25) is a liposomal vaccine containing a 25-amino-acid MUC1 peptide corresponding to the core peptide of MUC1 (STAPPAHGVTSAPDTRPAPGSTAPP) and coupled with a palmitoyl lysine residue at the C terminus which increases incorporation of this lipopeptide into the liposome particle [74, 75]. This strategy is mostly being used in nonsmall-cell lung carcinoma (NSCLC) and is well tolerated [62, 76]. Even though no immunological response was observed, this vaccine seems to have enhanced patient survival for stage IIIB/IV NSCLC in a phase II clinical trial [63]. A phase III study is in progress to confirm this result [64].

Another major strategy is TG4010, from Transgene SA, which is a recombinant virus of the Modified Vaccinia Ankara (MVA), encoding MUC1 and IL-2 (MVA MUC1-IL2). Unlike the L-BLP25 which is being used on NSCLC only, TG4010 is being used against several types of cancer, including prostate cancer, renal cell carcinoma (RCC), and NSCLC [58–60]. In prostate cancer, evidence of biological activity of the vaccine has been observed, with improved prostate-specific antigen (PSA) level doubling time [59]. In RCC, preliminary results were encouraging showing that this vaccine is well tolerated and indicating some evidence of MUC1-specific CD4+ and CD8+ T-cell responses [60]. The best clinical responses using TG4010 were observed for lung cancer. Indeed, TG4010 enhanced the effect of chemotherapy, with an increase in six-month, progression-free survival for patients who received chemotherapy in combination with the vaccine as compared with patients given chemotherapy only [61]. A phase IIB/III trial is currently being performed to evaluate the clinical advantage of combining chemotherapy plus TG4010 in NSCLC.

Encouraging results were also obtained in a pilot phase III clinical trial where early-stage breast cancer patients (stage II) were immunized with oxidized-mannan MUC1 [57]. No resurgence of the disease was observed in the sixteen patients who received the vaccine, whereas four patients among the fifteen who received a placebo relapsed.

Passive vaccination strategies are not the only way to exploit MUC1 T-cells response for immunotherapy. Adoptive immunotherapy (AIT) is another approach, which consists in the purification of patient PBMC (lymphocytes, dendritic cells in particular), followed by *ex vivo* stimulation of T cells and/or dendritic cell loaded with MUC1 peptides. These cells are then adoptively transferred back to the patients. Interest in this strategy is the ability of these T cells to kill patient tumor cells and the capacity of dendritic cells to enhance this T-cell response. The first clinical trial of MUC1 adoptive T-cells transfers showed no clinical response [77]. It was followed by combining the transfer of activated T lymphocytes with MUC1 peptides pulsed dendritic cells. This second clinical trial showed interesting clinical effects with one patient complete response [68]. At the same time, *Lepisto and colleague* reported that transfers of MUC1 peptide-pulsed dendritic cells can elicit specific MUC1 CD8+ and CD4+ response but did not induce benefit for patient survival [78]. All together, these studies suggest the need of combining T-cells and dendritic cells transfers to obtain optimal response in patients. In another study, Wright and colleague showed that tumor burden influences the quality of adoptively transferred MUC1 specific CTL. Indeed, CTL prepared from PBMC of treated breast cancer patients with no evidence of disease can generate CTL that kill cancer cells and produce type 1 cytokine. Inversely, CTL obtained from patients with macroscopic disease were infective [79]. More recently, it was shown that TH1 CD4+ lymphocytes transfers in combination with IFN-*γ* and IL-10 were effective to induce clinical response that enhances patient survival [69]. All these studies show the interest of adoptive transfers strategy for MUC1-based immunotherapy. However, the adoptive transfers approach needs *ex vivo* manipulation of cells which could be complex to perform compared to other passive vaccination strategies.

Behind these clinical trials which have reached phase III, many other MUC1 vaccines are being used to explore other ways of inducing specific MUC1 T-cell responses in breast, prostate, ovarian, pancreatic, and lung cancer, as summarized in Table 2. Furthermore, numerous studies have described MUC1 as an oncogenic protein, advocating the development of pharmacological studies to counteract this protein. This is the case for toxins which target the cytoplasmic tail (see Figure 1) of MUC1 [80, 81] or molecules that downregulate MUC1 protein [82, 83]. Both pharmacological and immunotherapeutic strategies based on MUC1 to treat cancer should now be actively pursued.

## Figures and Tables

**Table 1:** Strategies for the induction of anti-MUC1 T cell responses that are being studied *in vitro *and* in vivo*.

Author	Strategy	Cancer Model	Effect
Deguchi et al. [43]	*α*-gal epitope to increase immunogenicity of MUC1	Pancreatic cancer mouse model	Antibody induction, mouse tumor regression, induction of T cell responses
Kovjazin et al. [44]	ImMucin peptide 21mer	Mouse/PBMC of patients	CD4+ and CD8+ T lymphocyte responses *in vitro* and *in vivo *
Choi et al. [45]	DNA vaccine (MUC1/HSP70)	B16 mice	cytotoxic T cell response induction/ tumor growth inhibition
Chen et al. [46]	MUC1 mRNA, dendritic cell transfection	Pancreatic cancer	MUC1 mRNA-transfected dendritic cells can induce MUC1-specific CD8+ T cell responses
Wright et al. [47]	MUC1 peptide with substitution of O-glycosylation site	Human adenocarcinoma	O-glycosylation site substitution improves immunogenicity
Kobukai et al. [48]	MPA11P vehicle of a 30mer MUC1 peptide	Mouse	Reduction of tumor size, lymphocyte infiltration
Lakshmiarayanan et al. [49]	Tripartite MUC1 vaccine (TLR2, Thelpher, MUC1 glycopeptides)	Mouse model of mammary cancer	IgG antibodies, cytotoxic T lymphocytes, activation of innate immune response
Sugiura et al. [50]	MUC1 DNA vaccine	Mouse/colon	Induction of CD4+ T cell responses, not CD8+
Ryan et al. [39, 40]	TN MUC1 glycopeptide	Mouse	T cell responses against glycosylated peptides, but not unglycosylated peptides
Choi et al. [51]	MUC1 DNA vaccination, enhanced by mANT2 shRNA	Mouse melanoma	Combination enhanced effects of DNA vaccination, MUC1 CD8+ T cell responses
Jeon et al. [52]	DNA vaccination	Mouse	Tumor growth inhibition, CD8+ IFN-*γ* increased

**Table 2:** MUC1-based immunotherapy trials.

Author	Strategy	Clinical trial phase	Major observation	Cancer type
Apostolopouls et al. [57]	Oxidized Mannan-MUC1	III	Breast cancer recurrence prevention	Breast
Ramlau et al. [58]	TG4010	II	TG4010 can be coupled with chemotherapy	Lung
Dreicer et al. [59]	TG4010	II	Increased PSA doubling time	Prostate
Oudard et al. [60]	TG4010	II	MUC1 T cell responses	RCC
Quoix et al. [61]	TG4010	IIB	Improved survival	Lung
Ohyanagi et al. [62]	BLP25	I/II	Well tolerated, low side effects	Lung
Butts et al. [63]	BLP25	II/B	Increased survival	Lung
WU et al. [64]	BLP25	III inspire	In progress/increased survival	Lung
Butts et al. [65]	BLP25	I/II	New formulation well tolerated	Lung
Wright et al. [66]	MUC1 TIL Transfers	I/II	Influence of the tumor burden on adoptive transfer of MUC1 specific T cells	Breast
Lepisto et al. [67]	Dendritic cells pulsed with MUC1	I/II	Well tolerated, induction of T cell responses	Pancreas
Kondo et al. [68]	Dendritic cells and CTL transfer	I	Clinical response	Pancreas
Dobrzanski et al. [69]	Adoptive transfer CD4 T cells plus IL-10	I	Clinical response	Ovarian
Mohebtash et al. [70]	PANVAC-VF	II	Clinical effects	Breast/ovary
Ibrahim et al. [71]	AS1402 + Letrosole	II	Use of Letrosole uncompatible with AS1402 strategy	Breast
Pegram et al. [72]	AS1402	I	Well tolerated, need phase II to evaluate efficacy	Breast
Rittig et al. [73]	ARN muc1	I/II	Induction of CD4+ T cell responses	RCC

## Abbreviations

MPM: Malignant pleural mesothelioma
MUC1: Mucin1
TAA: Tumor associated antigen.
